# Supplementary material for: Investigating the Eye as a Biomarker of Gulf War Illness: Sphingolipid and Eicosanoid Composition in Tears and Plasma
Source: Biomolecules. 2025 Dec 10;15(12):1716. doi: 10.3390/biom15121716 (PMC12730409; doi:10.3390/biom15121716)
Supplement: Supplementary file 1 [file biomolecules-15-01716-s001.zip › biomolecules-3977787-supplementary.pdf]

Supplementary Materials

Table S1. SCIEX 5500 QQQ mass spectrometer settings for reverse phase chromatographic separation of sphingolipid species

Analyte ID	Q1 Mass (Da)	Q2 Mass (Da)	DP (volt)	EP (volt)	CE (volt)	CXP (volt)
d17:1 So	286.4	268.3	120	10	15	10
d17:0 Sa	288.4	270.4	120	10	21	10
d18:1 So	300.5	282.3	120	10	21	10
d18:0 Sa	302.5	284.3	120	10	21	10
d17:1 So1P	366.4	250.4	120	10	23	10
d17:0 Sa1P	368.4	252.4	120	10	23	10
d18:1 So1P	380.4	264.4	120	10	25	10
d18:0 Sa1P	382.4	266.4	120	10	25	10
Cer(de18:1/12:0)	482.6	264.4	80	10	41	10
Cer(de18:1/14:0)	510.7	264.4	80	10	43.5	10
Cer(de18:1/16:0)	538.7	264.4	80	10	46	10
Cer(de18:1/18:1)	564.7	264.4	80	10	48.5	10
Cer(de18:1/18:0)	566.7	264.4	80	10	48.5	10
Cer(de18:1/20:0)	594.7	264.4	80	10	51	10
Cer(de18:1/22:0)	622.8	264.4	80	10	53.5	10
Cer(de18:1/24:1)	648.9	264.4	80	10	56	10
Cer(de18:1/24:0)	650.9	264.4	80	10	56	10
Cer(de18:1/26:1)	676.9	264.4	80	10	58.5	10
Cer(de18:1/26:0)	678.9	264.4	80	10	58.5	10
C1P(de18:1/12:0)	562.4	264.4	80	10	41	10
C1P(de18:1/14:0)	590.4	264.4	80	10	43.5	10
C1P(de18:1/16:0)	618.5	264.4	80	10	46	10
C1P(de18:1/18:1)	644.5	264.4	80	10	48.5	10
C1P(de18:1/18:0)	646.5	264.4	80	10	48.5	10
C1P(de18:1/20:0)	674.4	264.4	80	10	51	10
C1P(de18:1/22:0)	702.7	264.4	80	10	53.5	10
C1P(de18:1/24:1)	728.6	264.4	80	10	56	10
C1P(de18:1/24:0)	730.6	264.4	80	10	56	10
C1P(de18:1/26:1)	756.7	264.4	80	10	58.5	10
C1P(de18:1/26:0)	758.7	264.4	80	10	58.5	10
MonHex(de18:1/12:0)	644.6	264.4	80	10	41	10
MonHex(de18:1/14:0)	672.6	264.4	80	10	43.5	10
MonHex(de18:1/16:0)	700.7	264.4	80	10	46	10
MonHex(de18:1/18:1)	726.7	264.4	80	10	48.5	10
MonHex(de18:1/18:0)	728.7	264.4	80	10	48.5	10
MonHex(de18:1/20:0)	756.7	264.4	80	10	51	10
MonHex(de18:1/22:0)	784.8	264.4	80	10	53.5	10
MonHex(de18:1/24:1)	810.9	264.4	80	10	56	10
MonHex(de18:1/24:0)	812.9	264.4	80	10	56	10
MonHex(de18:1/26:1)	838.9	264.4	80	10	58.5	10
MonHex(de18:1/26:0)	840.9	264.4	80	10	58.5	10
SM(de18:1/12:0)	647.7	184.4	80	10	41	10

SM(de18:1/14:0)	675.7	184.4	80	10	43.5	10
SM(de18:1/16:0)	703.8	184.4	80	10	46	10
SM(de18:1/18:1)	729.8	184.4	80	10	48.5	10
SM(de18:1/18:0)	731.8	184.4	80	10	48.5	10
SM(de18:1/20:0)	759.9	184.4	80	10	51	10
SM(de18:1/22:0)	787.9	184.4	80	10	53.5	10
SM(de18:1/24:1)	813.9	184.4	80	10	56	10
SM(de18:1/24:0)	815.9	184.4	80	10	56	10
SM(de18:1/26:1)	841.9	184.4	80	10	58.5	10
SM(de18:1/26:0)	843.9	184.4	80	10	58.5	10

DP: declustering potential; CE: collision energy; EP: entrance potential; CXP: cell exit potential. Transitions and settings were determined manually via direct infusion of all commercially available standards (Avanti Polar Lipids and Cayman Chemical). Settings and transitions were chosen based on the best signal obtained during infusion using manual tuning in SCIEX Analyst software.

Table S2. SCIEX 5500 QQQ mass spectrometer settings for reverse phase chromatographic separation of eicosanoid species

Analyte ID	Q1 Mass (Da)	Q2 Mass (Da)	DP (volt)	EP (volt)	CE (volt)	CXP (volt)
6keto PGF1α-d4	373.2	167	-80	-14	-33	-15
6-keto PGF1α	369.2	163	-80	-14	-33	-15
8-iso PGF2α-d4	357.4	197.1	-150	-13	-33	-14
8-iso PGF2α	353.4	193	-130	-13	-31	-11
TXB2-d4	373.2	173	-80	-13	-23	-15
TXB2	369.2	169	-80	-13	-25	-15
5-iPF2α-VI-d11	364.2	115	-90	-12	-28	-20
5-iPF2α-VI	353.2	114.9	-90	-12	-28	-16
PGE2-d9	360.2	280.3	-80	-13	-24	-12
PGE2	351.2	271.2	-80	-13	-25	-12
PGF2α-d9	362.2	193	-70	-10	-35	-18
PGF2α	353.2	193	-70	-10	-31	-18
PGD2-d9	360.2	280.3	-80	-10	-24	-12
PGD2	351.2	271.2	-80	-10	-23	-12
RvD3-d5	380.2	147	-68	-10	-24	-11
RvD3	375.2	147	-68	-10	-24	-11
RvD2-d5	380.2	141	-80	-12	-21	-10
RvD2	375.2	141	-80	-12	-22	-10
PGE1-d4	357.2	239	-105	-13	-20	-19
PGE1	353.2	235	-105	-13	-19	-19
RvD1-d5	380.2	141	-70	-13	-20	-10
RvD1	375.2	141	-70	-13	-22	-10
Lipoxin A4-d5	356.3	114.9	-90	-14	-21	-20
Lipoxin A4	351.3	114.8	-90	-13	-20	-15
PGA2-d4	337.2	275.3	-80	-14	-19	-12
PGA2	333.2	271.2	-80	-14	-19	-12
LTD4-d5	500.3	177	-105	-10	-24	-16
LTD4	495.3	176.9	-105	-10	-19	-14
LTC4-d5	629.3	272.1	-50	-13	-30	-13

LTC4	624.3	272.1	-60	-13	-30	-13
LTE4-d5	443.2	338.3	-80	-10	-24	-15
LTE4	438.2	333.1	-80	-10	-23	-15
LTB4-d4	339.2	197	-95	-14	-21	-16
LTB4	335.2	195	-95	-14	-22	-16
Maresin 2-d5	364.2	221.1	-65	-14	-16	-11
Maresin 2	359.2	221.1	-65	-14	-16	-11
(±)14,15-DHET-d11	348.2	207	-110	-14	-24	-14
(±)14,15-DHET	337.2	207	-110	-14	-26	-14
15-deoxy-Δ12,14-PGJ2-d4	319.2	275.3	-100	-9	-22	-10
15-deoxy-Δ12,14-PGJ2	315.2	271.2	-100	-9	-21	-10
(±)11,12-DHET-d11	348.2	167	-85	-12	-25	-14
(±)11,12-DHET	337.2	167	-85	-12	-24	-14
(±)8,9-DHET-d11	348.2	185.2	-93	-9	-23	-13
(±)8,9-DHET	337.2	185.2	-95	-10	-20	-13
20-HETE-d6	325.2	281.3	-85	-13	-21	-12
20-HETE	319.2	275.2	-85	-13	-21	-12
15 HETE-d8	327.2	226	-116	-13	-16	-16
15 HETE	319.2	219	-116	-13	-19	-16
12 HETE-d8	327.2	184.1	-90	-13	-20	-16
12 HETE	319.2	178.9	-90	-13	-19	-16
(±)14(15)-EET-d11	330.2	219.1	-90	-13	-15	-15
(±)14(15)-EET	319.2	219.1	-90	-13	-15	-15
5 HETE d8	327.2	116	-90	-13	-18	-10
5 HETE	319.2	115	-90	-13	-20	-10
(±)8(9)-EET-d11	330.2	123	-90	-12	-18	-11
(±)8(9)-EET	319.2	123	-90	-12	-18	-11
EPA d5	306.2	262.3	-86	-10	-15	-18
EPA	301.2	257.1	-86	-10	-17	-18
DHA-d5	332.2	288.3	-95	-12	-14	-12
DHA	327.2	283.2	-95	-12	-19	-12
AA-d8	311.2	267.3	-150	-13	-18	-16
AA	303.2	259.2	-150	-13	-17	-14
DHGLA-d6	311.2	267.2	-105	-14	-20	-13
DHGLA	305.2	261.2	-90	-10	-43	-16

DP: declustering potential; CE: collision energy; EP: entrance potential; CXP: cell exit potential. Transitions and settings were determined manually via direct infusion of all commercially available standards (Cayman Chemical). Settings and transitions were chosen based on the best signal obtained during infusion using manual tuning in SCIEX Analyst software.

Table S3. Complete tear biomarker data, including non-significant species, in GWI cases and controls

**Biomarkers**	**GWI Mean (SD) (n=19)**	**Control Mean (SD) (n=21)**	**p-value**	**Adjusted *p*-value (FDR)**
**Tear Sphingolipids (pmol)**				
Cer C14:0	0.33 (0.30)	0.16 (0.10)	0.046	0.23

Cer C16:0	12.52 (13.49)	5.30 (5.08)	0.06	0.25
Cer C16:0 DH	0.33 (0.33)	0.19 (0.16)	0.22	0.36
Cer C18:1	0.46 (0.50)	0.26 (0.18)	0.27	0.36
Cer C18:0	1.18 (1.34)	0.64 (0.53)	0.26	0.36
Cer C20:0	1.04 (1.14)	0.58 (0.50)	0.26	0.36
Cer C22:0	2.51 (2.71)	1.21 (1.26)	0.08	0.29
Cer C24:1	2.40 (2.46)	1.10 (1.21)	0.07	0.27
Cer C24:0	3.70 (3.75)	1.75 (1.49)	0.04	0.26
Cer C26:1	0.17 (0.19)	0.08 (0.08)	0.057	0.26
Cer C26:0	0.95 (0.89)	0.64 (0.33)	0.39	0.44
Total Cer	25.60 (26.47)	11.89 (10.39)	0.08	0.26
MHC C14:0	0.11 (0.14)	0.06 (0.05)	0.22	0.36
MHC C16:0	0.83 (0.82)	0.49 (0.38)	0.11	0.34
MHC C16:0 DH	0.53 (0.98)	0.11 (0.14)	0.08	0.29
MHC C18:1	0.02 (0.01)	0.01 (0.01)	0.14	0.44
MHC C18:0	0.10 (0.08)	0.05 (0.03)	0.03	0.19
MHC C20:0	0.06 (0.05)	0.04 (0.02)	0.13	0.19
MHC C22:0	0.24 (0.20)	0.12 (0.07)	0.02	0.17
MHC C24:1	0.26 (0.30)	0.11 (0.12)	0.057	0.47
MHC C24:0	0.42 (0.39)	0.20 (0.11)	0.02	0.55
MHC C26:1	0.00 (0.00)	0.00 (0.00)	N/A	N/A
MHC C26:0	0.03 (0.04)	0.01 (0.01)	0.52	0.41
Total MHC	2.59 (2.90)	1.19 (0.78)	0.05	0.26
SM C14:0	2.85 (2.55)	1.52 (1.23)	0.05	0.27
SM C16:0	50.23 (56.89)	26.78 (22.87)	0.13	0.36
SM C16:0 DH	4.10 (4.86)	2.07 (1.75)	0.11	0.36
SM C18:1	0.55 (0.58)	0.38 (0.40)	0.26	0.56
SM C18:0	7.04 (8.82)	4.53 (4.23)	0.37	0.55
SM C20:0	8.07 (10.68)	4.31 (3.86)	0.36	0.42
SM C22:0	13.52 (16.13)	7.06 (5.99)	0.20	0.36
SM C24:1	12.15 (16.16)	7.41 (6.69)	0.34	0.53
SM C24:0	18.12 (21.16)	8.64 (6.61)	0.08	0.30
SM C26:1	1.04 (1.11)	0.52 (0.42)	0.07	0.29
SM C26:0	2.08 (2.55)	1.04 (0.79)	0.15	0.36
Total SM	119.75 (138.73)	64.26 (53.68)	0.21	0.36
C16-C1P	0.26 (0.31)	0.16 (0.14)	0.21	0.49
Sph	13.93 (15.12)	30.24 (77.65)	0.85	0.55
SPL Total	162.13 (164.90)	107.73 (113.96)	0.15	0.53
**Tear Sphingolipids (mol %l)**				
Cer C14:0	1.46 (0.43)	1.59 (0.59)	0.43	0.63

Cer C16:0	46.86 (9.85)	41.94 (8.23)	0.07	0.28
Cer C16:0 DH	1.31 (0.52)	1.72 (0.56)	0.01	0.23
Cer C18:1	2.05 (1.03)	2.89 (1.38)	0.04	0.25
Cer C18:0	4.42 (1.14)	5.45 (1.15)	0.01	0.17
Cer C20:0	4.02 (1.14)	4.88 (0.82)	0.01	0.17
Cer C22:0	9.48 (1.85)	9.45 (2.06)	0.96	0.94
Cer C24:1	9.06 (2.61)	7.76 (2.72)	0.15	0.42
Cer C24:0	15.48 (3.91)	16.00 (3.86)	0.69	0.72
Cer C26:1	0.66 (0.35)	0.60 (0.33)	0.58	0.78
Cer C26:0	5.20 (3.33)	7.72 (4.02)	0.03	0.22
Cer% of Total	14.76 (4.75)	12.02 (3.88)	0.06	0.06
MHC C14:0	4.39 (2.44)	4.68 (1.94)	0.80	0.84
MHC C16:0	34.59 (6.74)	38.93 (6.46)	0.05	0.28
MHC C16:0 DH	13.20 (9.11)	9.26 (8.04)	0.19	0.45
MHC C18:1	0.77 (0.50)	0.91 (0.49)	0.28	0.53
MHC C18:0	4.32 (1.31)	4.50 (1.45)	0.69	0.92
MHC C20:0	3.05 (1.38)	3.43 (1.05)	0.13	0.69
MHC C22:0	10.66 (2.31)	9.99 (2.02)	0.36	0.45
MHC C24:1	9.74 (2.33)	9.14 (3.04)	0.51	0.80
MHC C24:0	18.40 (4.66)	17.93 (4.67)	0.76	0.88
MHC C26:1	0.00 (0.00)	0.00 (0.00)	N/A	N/A
MHC C26:0	0.87 (0.50)	1.22 (0.73)	0.10	0.25
MHC % of Total	1.77 (1.18)	1.82 (1.33)	0.89	0.74
SM C14:0	2.93 (1.38)	2.62 (0.62)	0.89	0.57
SM C16:0	40.01 (11.22)	41.24 (5.19)	0.75	0.85
SM C16:0 DH	3.44 (0.76)	3.18 (0.38)	0.15	0.42
SM C18:1	0.56 (0.34)	0.56 (0.35)	0.94	0.92
SM C18:0	5.71 (1.14)	6.48 (1.32)	0.09	0.28
SM C20:0	6.10 (1.41)	6.21 (1.00)	0.79	0.85
SM C22:0	11.38 (2.51)	10.98 (1.48)	0.80	0.71
SM C24:1	10.85 (3.52)	10.92 (2.22)	0.96	0.91
SM C24:0	16.33 (4.81)	15.06 (3.60)	0.52	0.62
SM C26:1	0.93 (0.26)	0.86 (0.27)	0.44	0.77
SM C26:0	1.77 (0.48)	1.89 (0.87)	0.99	0.62
SM% of Total	67.79 (18.06)	64.48 (18.66)	0.62	0.65
**Tear Eicosanoids (pmol)**				
TXB2	0.002 (0.004)	0.001 (0.001)	0.31	0.36
PGE2	0.01 (0.01)	0.004 (0.003)	0.04	0.19
PGF2α	0.01 (0.004)	0.01 (0.003)	0.90	0.94
PGD2	0.04 (0.08)	0.02 (0.02)	0.92	0.41

Resolvin D1	0.04 (0.02)	0.04 (0.02)	0.79	0.87
PGA2	0.07 (0.03)	0.06 (0.03)	0.57	0.72
(±)14,15-DHET	0.01 (0.01)	0.01 (0.004)	0.07	0.32
(±)11,12-DHET	0.01 (0.01)	0.01 (0.01)	0.12	0.57
(±)8,9-DHET	0.002 (0.002)	0.002 (0.002)	0.10	0.48
20-HETE	1.97 (1.03)	2.33 (1.45)	0.37	0.57
15-HETE	0.12 (0.15)	0.04 (0.04)	0.02	0.19
12-HETE	0.09 (0.09)	0.07 (0.06)	0.54	0.57
(±)14(15)-EET	0.04 (0.03)	0.01 (0.01)	0.002	0.04
5-HETE	0.17 (0.15)	0.12 (0.10)	0.15	0.41
5-OxoETE	0.38 (0.35)	0.20 (0.21)	0.02	0.25
(±)8(9)-EET	0.03 (0.03)	0.01 (0.01)	0.008	0.09
EPA	1.38 (2.66)	0.36 (0.36)	0.07	0.29
DHA	2.73 (2.49)	1.41 (1.15)	0.06	0.22
AA	29.83 (22.64)	15.70 (15.93)	0.02	0.19
DHGLA	0.21 (0.38)	0.11 (0.16)	0.89	0.48

GWI = Gulf War Illness; SD = standard deviation; Cer = Ceramide (chain length indicated by Cxx and saturation status by :x, where 0 = saturated and 1 = monoun-saturated); MHC = Monohexosylceramide (chain length indicated by Cxx and saturation status by :x, where 0 = saturated and 1 = monoun-saturated); SM = Sphingomyelin (chain length indicated by Cxx and saturation status by :x, where 0 = saturated and 1 = monoun-saturated); C1P = Ceramide-1-phosphate; Sph = Sphingosine; DH = dihydro form of the lipid species (saturated sphingoid base). TXB2 = Thromboxane B2; PGE2 = Prostaglandin E2; PGF2α = Prostaglandin F2 Alpha; PGD2 = Prostaglandin D2; PGA2 = Prostaglandin A2; (±)14,15-DHET = 14,15-Dihydroxyeicosatrienoic acid; (±)11,12-DHET = 11,12-Dihydroxyeicosatrienoic acid; (±)8,9-DHET = 8,9-Dihydroxyeicosatrienoic acid; 20-HETE = 20-Hydroxyeicosatetraenoic acid; 15-HETE = 15-Hydroxyeicosatetraenoic acid; 12-HETE = 12-Hydroxyeicosatetraenoic acid; (±)14(15)-EET = 14(15)-Epoxyeicosatrienoic acid; 5-HETE = 5-Hydroxyeicosatetraenoic acid; 5-OxoETE = 5-Oxo-Eicosatetraenoic acid; (±)8(9)-EET = 8(9)-Epoxyeicosatrienoic acid; EPA = Eicosapentaenoic acid; DHA = Docosahexaenoic acid; AA = Arachidonic acid; DHGLA = Dihomo-γ-linolenic acid. pmol = picomoles; mol % = mole percent. Raw $p < 0.05$ was considered statistically significant. Adjusted p-values were calculated using the Benjamini–Hochberg procedure to control the false discovery rate (FDR = 10%). Variables with adjusted $p \leq 0.10$ were considered significant. N/A indicates that a p-value was not available for that variable due to missing or excluded data.

Table S4. Complete plasma biomarker data, including non-significant species, in GWI cases and controls

Biomarkers	GWI Mean (SD) (n=19)	Control Mean (SD) (n=21)	p-value	Adjusted *p*-value (FDR)
**Plasma Sphingolipids (pmol)**				
Cer C14:0	2.11 (0.73)	2.49 (0.87)	0.15	0.36
Cer C16:0	13.61 (5.25)	16.65 (5.57)	0.08	0.85
Cer C16:0 DH	0.77 (0.36)	0.78 (0.53)	0.67	0.97

Cer C18:1	0.64 (0.26)	0.68 (0.41)	0.74	0.85
Cer C18:0	8.82 (3.12)	11.84 (4.39)	0.02	0.18
Cer C20:0	20.83 (6.58)	26.16 (9.49)	0.048	0.25
Cer C22:0	212.68 (59.23)	232.99 (70.75)	0.33	0.55
Cer C24:1	162.06 (59.28)	202.23 (80.03)	0.09	0.47
Cer C24:0	715.84 (242.13)	878.16 (272.08)	0.047	0.55
Cer C26:1	7.37 (3.26)	8.05 (2.92)	0.35	0.61
Cer C26:0	11.02 (6.66)	12.17 (4.29)	0.16	0.69
Total Cer	1155.76 (319.22)	1392.19 (402.00)	0.048	0.25
MHC C14:0	1.89 (0.72)	1.57 (0.63)	0.14	0.36
MHC C16:0	84.53 (25.32)	87.43 (32.94)	0.76	0.76
MHC C16:0 DH	0.59 (0.35)	0.51 (0.22)	0.77	0.59
MHC C18:1	1.05 (0.31)	1.01 (0.40)	0.68	0.81
MHC C18:0	3.79 (0.94)	3.44 (1.17)	0.30	0.53
MHC C20:0	6.03 (2.08)	5.99 (2.14)	0.85	0.97
MHC C22:0	144.77 (56.15)	127.90 (55.70)	0.35	0.55
MHC C24:1	94.68 (35.46)	87.36 (40.47)	0.20	0.81
MHC C24:0	239.39 (123.95)	187.51 (91.59)	0.21	0.57
MHC C26:1	0.00 (0.00)	0.00 (0.00)	N/A	N/A
MHC C26:0	2.55 (2.06)	2.06 (1.10)	0.63	0.55
Total MHC	579.26 (226.19)	504.78 (213.49)	0.29	0.52
SM C14:0	909.25 (84.45)	1155.35 (389.08)	0.054	0.14
SM C16:0	481.34 (110.38)	532.51 (113.13)	0.15	0.92
SM C16:0 DH	537.32 (68.77)	622.58 (127.11)	0.01	0.17
SM C18:1	505.89 (101.63)	567.97 (160.51)	0.16	0.36
SM C18:0	857.70 (148.75)	797.62 (176.83)	0.26	0.48
SM C20:0	1334.88 (229.70)	1152.93 (157.26)	0.005	0.09
SM C22:0	2175.69 (452.10)	1911.47 (262.31)	0.03	0.19
SM C24:1	2382.55 (452.15)	2564.47 (521.76)	0.25	1.00
SM C24:0	2053.64 (358.19)	1984.45 (331.67)	0.53	0.53
SM C26:1	62.87 (17.93)	59.59 (17.15)	0.56	0.72
SM C26:0	38.19 (9.75)	39.24 (10.13)	0.74	0.85
Total SM	11339.32 (1525.88)	11388.17 (1742.04)	0.93	0.96
C16-C1P	76.57 (31.91)	82.63 (30.86)	0.55	0.71
Sph	81.77 (74.54)	75.96 (57.74)	0.92	0.87
S1P	83.27 (40.43)	82.67 (37.54)	0.96	0.97
SPL Total	13315.96 (1518.34)	13526.40 (2020.03)	0.71	0.84
**Plasma Sphingolipids (mol %)**				
Cer C14:0	0.19 (0.05)	0.18 (0.05)	0.83	0.90
Cer C16:0	1.27 (0.67)	1.24 (0.45)	1.00	1.00

Cer C16:0 DH	0.07 (0.04)	0.06 (0.06)	0.17	0.67
Cer C18:1	0.06 (0.04)	0.05 (0.03)	0.49	0.41
Cer C18:0	0.82 (0.38)	0.88 (0.35)	0.50	0.78
Cer C20:0	1.86 (0.55)	1.90 (0.54)	0.82	0.90
Cer C22:0	18.47 (2.33)	16.78 (2.30)	0.01	0.19
Cer C24:1	14.52 (5.01)	14.52 (4.21)	1.00	1.00
Cer C24:0	61.14 (6.76)	62.92 (5.52)	0.37	0.70
Cer C26:1	0.65 (0.21)	0.58 (0.15)	0.26	0.48
Cer C26:0	0.94 (0.41)	0.88 (0.22)	0.79	0.71
Cer% of Total	8.77 (2.67)	10.30 (2.52)	0.07	0.27
MHC C14:0	0.33 (0.09)	0.32 (0.06)	0.46	0.64
MHC C16:0	15.14 (2.28)	17.89 (2.78)	0.002	0.04
MHC C16:0 DH	0.10 (0.03)	0.12 (0.07)	0.85	0.57
MHC C18:1	0.20 (0.06)	0.21 (0.06)	0.54	0.68
MHC C18:0	0.71 (0.23)	0.76 (0.32)	0.73	0.78
MHC C20:0	1.10 (0.29)	1.24 (0.22)	0.10	0.30
MHC C22:0	25.17 (4.10)	25.15 (2.75)	0.98	0.98
MHC C24:1	17.11 (4.52)	17.65 (3.71)	0.68	0.68
MHC C24:0	39.72 (5.50)	36.28 (3.56)	0.02	0.36
MHC C26:1	0.00 (0.00)	0.00 (0.00)	N/A	N/A
MHC C26:0	0.41 (0.16)	0.40 (0.10)	0.63	0.92
MHC % of Total	4.41 (1.88)	3.74 (1.46)	0.31	0.42
SM C14:0	8.09 (0.75)	10.10 (2.76)	0.005	0.07
SM C16:0	4.24 (0.70)	4.67 (0.61)	0.048	0.25
SM C16:0 DH	4.75 (0.31)	5.44 (0.47)	<0.001	0.001
SM C18:1	4.47 (0.76)	4.95 (0.97)	0.09	0.29
SM C18:0	7.57 (0.93)	7.03 (1.26)	0.13	0.36
SM C20:0	11.74 (1.00)	10.19 (0.95)	<0.001	0.002
SM C22:0	19.05 (1.98)	16.87 (1.26)	<0.001	0.007
SM C24:1	21.08 (3.21)	22.39 (2.03)	0.05	0.36
SM C24:0	18.10 (1.90)	17.49 (2.02)	0.39	0.39
SM C26:1	0.56 (0.19)	0.52 (0.13)	0.57	0.57
SM C26:0	0.34 (0.08)	0.35 (0.09)	0.76	0.85
SM% of Total	85.01 (3.81)	84.17 (2.90)	0.44	0.62
**Plasma Eicosanoids**				
TXB2	1.23 (1.58)	1.26 (2.70)	0.27	0.98
PGE2	1.11 (1.22)	0.83 (0.98)	0.12	0.61
PGF2α	1.10 (1.40)	0.81 (0.62)	0.73	0.58
PGD2	5.04 (3.39)	4.45 (5.27)	0.16	0.81
Resolvin D1	4.99 (0.16)	5.01 (0.16)	0.73	0.85

PGA2	9.37 (0.51)	9.40 (0.48)	0.92	0.91
(±)14,15-DHET	1.47 (0.35)	1.58 (0.47)	0.75	0.61
(±)11,12-DHET	1.18 (0.30)	1.38 (0.30)	0.04	0.23
(±)8,9-DHET	0.97 (0.44)	1.25 (0.53)	0.08	0.28
20-HETE	126.43 (13.83)	124.64 (8.09)	0.62	0.77
15-HETE	10.68 (5.62)	13.77 (11.73)	0.41	0.53
12-HETE	27.43 (39.21)	36.96 (42.23)	0.19	0.64
(±)14(15)-EET	9.23 (4.19)	10.58 (3.50)	0.28	0.49
5-HETE	56.22 (26.04)	86.70 (104.80)	0.96	0.44
5-OxoETE	31.28 (13.30)	46.38 (29.92)	0.05	0.25
(±)8(9)-EET	2.85 (0.95)	3.01 (1.14)	0.62	0.77
EPA	615.82 (565.53)	511.28 (257.07)	0.67	0.63
DHA	1095.81 (891.52)	1168.49 (392.01)	0.10	0.85
AA	8140.96 (3675.77)	10450.75 (3926.34)	0.06	0.26
DHGLA	33.84 (47.35)	45.68 (61.42)	0.24	0.68

GWI = Gulf War Illness; SD = standard deviation; Cer = Ceramide (chain length indicated by Cxx and saturation status by :x, where 0 = saturated and 1 = monoun-saturated); MHC = Monohexosylceramide (chain length indicated by Cxx and saturation status by :x, where 0 = saturated and 1 = monoun-saturated); SM = Sphingomyelin (chain length indicated by Cxx and saturation status by :x, where 0 = saturated and 1 = monoun-saturated); C1P = Ceramide-1-phosphate; Sph = Sphingosine; DH = dihydro form of the lipid species (saturated sphingoid base). TXB2 = Thromboxane B2; PGE2 = Prostaglandin E2; PGF2α = Prostaglandin F2 Alpha; PGD2 = Prostaglandin D2; PGA2 = Prostaglandin A2; (±)14,15-DHET = 14,15-Dihydroxyeicosatrienoic acid; (±)11,12-DHET = 11,12-Dihydroxyeicosatrienoic acid; (±)8,9-DHET = 8,9-Dihydroxyeicosatrienoic acid; 20-HETE = 20-Hydroxyeicosatetraenoic acid; 15-HETE = 15-Hydroxyeicosatetraenoic acid; 12-HETE = 12-Hydroxyeicosatetraenoic acid; (±)14(15)-EET = 14(15)-Epoxyeicosatrienoic acid; 5-HETE = 5-Hydroxyeicosatetraenoic acid; 5-OxoETE = 5-Oxo-Eicosatetraenoic acid; (±)8(9)-EET = 8(9)-Epoxyeicosatrienoic acid; EPA = Eicosapentaenoic acid; DHA = Docosahexaenoic acid; AA = Arachidonic acid; DHGLA = Dihomo-γ-linolenic acid. pmol = picomoles; mol % = mole percent. Raw $p < 0.05$ was considered statistically significant. Adjusted p-values were calculated using the Benjamini–Hochberg procedure to control the false discovery rate (FDR = 10%). Variables with adjusted $p \leq 0.10$ were considered significant. N/A indicates that a p-value was not available for that variable due to missing or excluded data.
